# Underestimation of cardiovascular risk by QRISK3 and RA-adapted SCORE2 in a Chinese rheumatoid arthritis population

**DOI:** 10.3389/fcvm.2026.1712088

**Published:** 2026-02-12

**Authors:** Xinpei Li, Xiaowei Ni, Wenjuan Qian, Chen Sun, Xiaoling Yuan, Yan Zhang, Yabing Zhang, Zening Yuan

**Affiliations:** Department of Rheumatology and Immunology, The First People’s Hospital of Zhangjiagang City (Zhangjiagang Hospital Affiliated to Soochow University), Zhangjiagang, China

**Keywords:** cardiovascular disease, disease activity(DAS28), QRISK3 algorithm, RA-adapted SCORE2 algorithm, rheumatoid arthritis

## Abstract

**Background:**

Patients with rheumatoid arthritis (RA) face a substantially increased risk of cardiovascular disease (CVD), yet existing risk prediction models often perform poorly in this population. QRISK3 and RA-adapted SCORE2 incorporate RA in their frameworks, but their validity in Asian cohorts remains uncertain.

**Methods:**

We conducted a retrospective observational study using electronic hospital records from The First People's Hospital of Zhangjiagang City in China (2020–2025). Adults with confirmed RA who subsequently experienced a first major CVD event (coronary heart disease, ischemic stroke, or transient ischemic attack) were included. QRISK3 and RA-adapted SCORE2 were applied to the conventional thresholds of ≥10% and ≥5% respectively, to define high risk. Agreement between tools was assessed with Cohen's kappa and McNemar's test. Adjusted logistic regression examined demographic, RA-related, and traditional risk factors associated with risk underestimation.

**Results:**

A total of 249 patients with RA and CVD were included. Both tools substantially underestimated risk, with underestimation more frequent for QRISK3 than RA-adapted SCORE2. Agreement between the two was moderate (κ = 0.44), with discordance most marked across age, glucocorticoid exposure, and disease activity subgroups. Patients with high baseline DAS28 scores were particularly likely to be misclassified as low risk. In adjusted models, diabetes, chronic kidney disease, and systemic steroid use were associated with greater underestimation.

**Conclusions:**

QRISK3 and RA-adapted SCORE2substantially underestimated cardiovascular risk in Chinese patients with RA, especially those with active disease. European-derived tools may not be reliable in this setting, underscoring the need for recalibrated or RA-specific models.

## Introduction

Rheumatoid arthritis (RA) is a systemic autoimmune disease characterised by chronic inflammation and accelerated atherosclerosis, resulting in an increased burden of cardiovascular disease (CVD) (1). Past evidence has shown that patients with RA experience approximately 1.5 to 2 times higher rates of atherosclerotic CVD compared with the general population (2), and about half of deaths are related to CVD causes (3). This elevated burden arises from a combination of conventional risk factors, systemic inflammation, and genetic susceptibility.

Before most CVD prediction tools were developed and they incorporated traditional risk factors in the general population. Previous studies indicated that these tools systematically underestimate risk in patients with RA, reflecting the failure to account for disease-specific factors such as chronic inflammation and RA-related comorbidities (4). In response, some RA-specific CVD risk models have been proposed. For example, the modified SCORE algorithm, recommended by the European League Against Rheumatism (EULAR), applies a 1.5 multiplication factor to SCORE estimates in patients with defined RA characteristics (5). Similarly, QRISK3, derived from a large UK primary care database, incorporates RA and systemic lupus erythematosus as independent predictors, alongside conventional risk factors (6).

However, the performance of RA-adapted cardiovascular risk algorithms remains incompletely characterised, especially outside their original derivation settings. Asian populations, including China, typically have lower baseline CVD incidence and distinct risk factor distributions compared with Western cohorts (7). The Framingham equations and the ACC/AHA Pooled Cohort Equations (PCEs) have been evaluated in Asian populations, with notable underestimation in females (8–10). But the performance of RA-specific algorithms in these populations is limited. QRISK3 and RA-adapted SCORE2, developed and validated in European or UK cohorts, have not been systematically examined in Chinese patients with RA (11).

To address these gaps, we conducted a retrospective cohort study in China to evaluate the performance of QRISK3 and RA-adapted SCORE2 against observed CVD outcomes in patients with established RA. We examined how QRISK3 and RA-adapted SCORE2 classify cardiovascular risk in this population, quantified the proportion of patients classified above or below commonly used clinical thresholds, and explored patient characteristics associated with potential misclassification.

## Method

### Study design and setting

We carried out a retrospective observational study at Zhangjiagang First People's Hospital in China. Data were routinely collected from electronic hospital records, which included details from outpatient rheumatology visits, inpatient admissions, diagnostic reports, and laboratory results. The observation window was from 1 May 2020 to 31 May 2025. Ethical approval was obtained from the institutional review board, and individual patient consent was waived because all data were anonymised and analysed retrospectively.

### Study population

We retrospectively identified adult patients (≥18 years) with rheumatoid arthritis (RA) from electronic hospital records between 1 May 2020 and 31 May 2025. Within this RA cohort, we then ascertained patients who had a documented cardiovascular disease (CVD) diagnosis in the same records. Both RA and CVD diagnoses were confirmed by review of clinical documentation, serological findings, and specialist reports. Patients with a prior history of CVD or baseline statin use were excluded. From within this RA population, we included patients who had a clinician-confirmed diagnosis of CVD during the study period.

### Cardiovascular outcomes

In this study, cardiovascular outcomes were defined in line with the QRISK3 development studies. The composite endpoint comprised the first recorded occurrence of fatal or non-fatal coronary heart disease, ischemic stroke, or transient ischemic attack. Events were initially identified from hospital diagnosis records and then confirmed by detailed clinical review, which incorporated clinical summaries, relevant imaging, and cardiology reports.

### Data collection

Demographic, clinical, medication and laboratory information were extracted from hospital records. Demographic data included age, sex, and smoking status. Clinical data include the comorbidities history (diabetes, chronic kidney disease, atrial fibrillation, migraine, systemic lupus erythematosus, and severe mental illness) and RA-related data [baseline disease activity score (DAS28: Remission-low/moderate/high), rheumatoid factor and anti-cyclic citrullinated peptide antibody status]. Treatment information included the use of disease-modifying antirheumatic drugs, antihypertensive therapy, atypical antipsychotics, and treatment for erectile dysfunction. Physiological and laboratory measures comprised systolic blood pressure, within-person variation in systolic blood pressure (calculated as the standard deviation of the two most recent readings), total cholesterol, HDL cholesterol, cholesterol/HDL ratio, height, weight, and body mass index. Where baseline medical history or lifestyle information was incomplete, these data were updated or confirmed during the clinical assessment at the time of the cardiovascular diagnosis.

### Statistical analysis

Continuous variables were presented as means with standard deviations or medians with interquartile ranges, depending on distribution. Categorical variables were reported as counts and percentages.

Predicted risks were calculated for each patient using QRISK3 and RA-adapted SCORE2. QRISK3 estimates the 10-year risk of a first major cardiovascular event. Following the UK practice guideline, patients were classified as being at high risk if their predicted risk was 10% or greater. The RA-adapted SCORE2 estimate was calculated as the 10-year risk of cardiovascular death, with SCORE2 values multiplied by 1.5 to account for rheumatoid arthritis following the EULAR suggestion. And a threshold of 5% was used to define high risk. Concordance between QRISK3 and RA-adapted SCORE2 classifications was assessed using Cohen's kappa (12), and McNemar's test (13) for paired proportions.

This was a case-only study restricted to patients with rheumatoid arthritis who experienced a first cardiovascular event. Consequently, traditional model performance metrics such as discrimination (C-statistics) and calibration could not be assessed. Analyses therefore focused on threshold-based risk classification, examining whether patients with confirmed events would have been classified above or below commonly used clinical risk thresholds. Underestimation was defined in our cohort as cases in which patients with confirmed cardiovascular outcomes had predicted low risks. The primary analysis quantified the risk distribution and the proportion of underestimation by each tool. Subgroup analyses repeated this calculation within sex, age group (<50, ≥50 years), glucocorticoid exposure, baseline DAS28 score, type of cardiovascular outcome (coronary heart disease, ischemic stroke, transient ischemic attack), and time from RA diagnosis to cardiovascular event (≤10 years vs. >10 years. Crude and age- and sex-adjusted logistic regression models were used to examine patient characteristics, a range of demographic, RA-related, and traditional cardiovascular risk factors associated with underestimation. Results were presented as odds ratios (OR) with 95% confidence intervals (CI).

All analyses were conducted using R software (version 4.4.1, R Foundation for Statistical Computing, Vienna, Austria). Statistical significance was defined as a two-sided *p*-value of <0.05.

## Result

A total of 249 patients with RA and subsequent CVD were included, of whom 131 (52.6%) were female. The median age at RA diagnosis was 55 years (IQR, 51–60), with a median age at first CVD event of 64 years (IQR, 60–68). Current smoking was more common among men (36%) than women (8%). And 64% of patients had moderate baseline disease activity (DAS28). Coronary heart disease was the most frequent event, followed by ischemic stroke and transient ischemic attack. More information is reported in Table 1.

**Table 1:** Baseline characteristics of the study cohort.

Characteristic	Overall (*N* = 249)	Female (*n* = 131)	Male (*n* = 118)
RA diagnosis age	55 (51–60)	57 (54–63)	53 (48–57)
CVD age,	64 (60–68)	67 (63–71)	62 (58–65)
Smoking status, *n* (%)			
Never	138 (55)	91 (69)	47 (40)
Former	58 (23)	29 (22)	29 (25)
Current	53 (21)	11 (8.4)	42 (36)
Regular steroid use, *n* (%)	128 (51)	73 (56)	55 (47)
Blood pressure treatment, *n* (%)	76 (31)	40 (31)	36 (31)
Antihypertensive treatment, *n* (%)	65 (26)	27 (21)	38 (32)
Extra-articular disease, *n* (%)	44 (18)	21 (16)	23 (19)
Erosive disease, *n* (%)	57 (23)	29 (22)	28 (24)
DAS28 category, *n* (%)			
Remission/Low	49 (20)	23 (18)	26 (22)
Moderate	159 (64)	86 (66)	73 (62)
High	41 (16)	22 (17)	19 (16)
Hypertension, *n* (%)	70 (28)	36 (27)	34 (29)
Diabetes, *n* (%)	78 (31)	46 (35)	32 (27)
Obesity, *n* (%)	19 (7.6)	9 (6.9)	10 (8.5)
Hyperlipidaemia, *n* (%)	40 (16)	17 (13)	23 (19)
Atrial fibrillation, *n* (%)	11 (4.4)	6 (4.6)	5 (4.2)
Chronic kidney disease, *n* (%)	30 (12)	19 (15)	11 (9.3)
Chronic lung disease, *n* (%)	45 (18)	24 (18)	21 (18)
Chronic liver disease, *n* (%)	12 (4.8)	7 (5.3)	5 (4.2)

**Figure 1:**
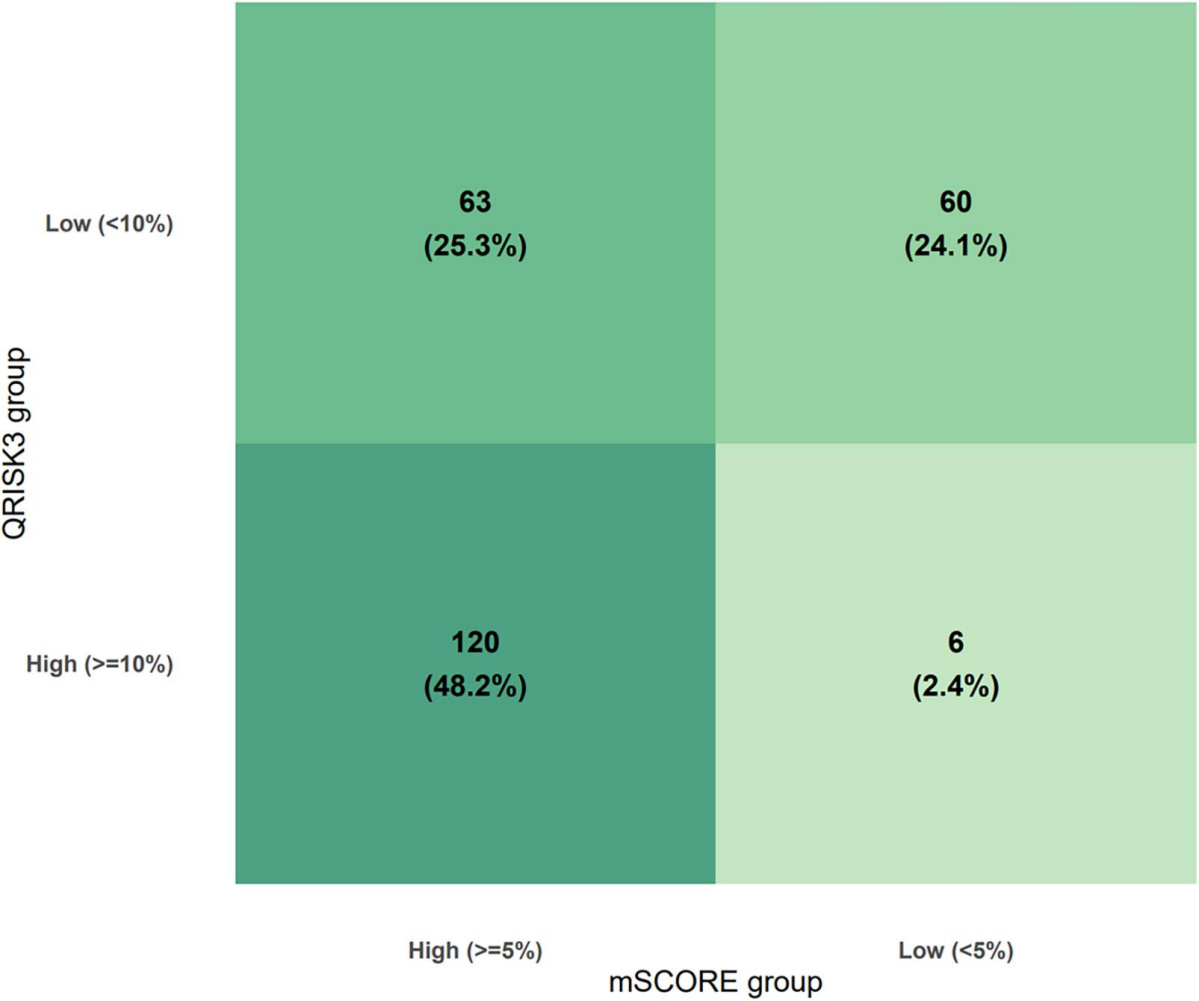
Cross-classification of RA patients by QRISK3 and RA-adapted SCORE2.

QRISK3 classified 126 patients (50.6%) as high risk (≥10%) and 123 (49.4%) as low risk (<10%). In contrast, RA-adapted SCORE2 identified 183 patients (73.5%) as high risk (≥5%) and 66 (26.5%) as low risk (<5%). Overall, QRISK3 underestimated cardiovascular risk in 123 patients (49.4%), while RA-adapted SCORE2 underestimated risk in 66 patients (26.5%). Cross-classification demonstrated substantial discordance between the tools in Figure 1.

Agreement between QRISK3 and RA-adapted SCORE2 classifications was moderate. Overall, the two tools assigned patients to the same risk category in 72.3% of cases. Cohen's *κ* was 0.44, indicating moderate agreement beyond chance (*p* < 0.001). McNemar's test showed significant asymmetry in discordant classifications (*χ*^2^ = 45.4, *p* < 0.001), with RA-adapted SCORE2 more frequently classifying patients as high risk compared with QRISK3.

**Figure 2:**
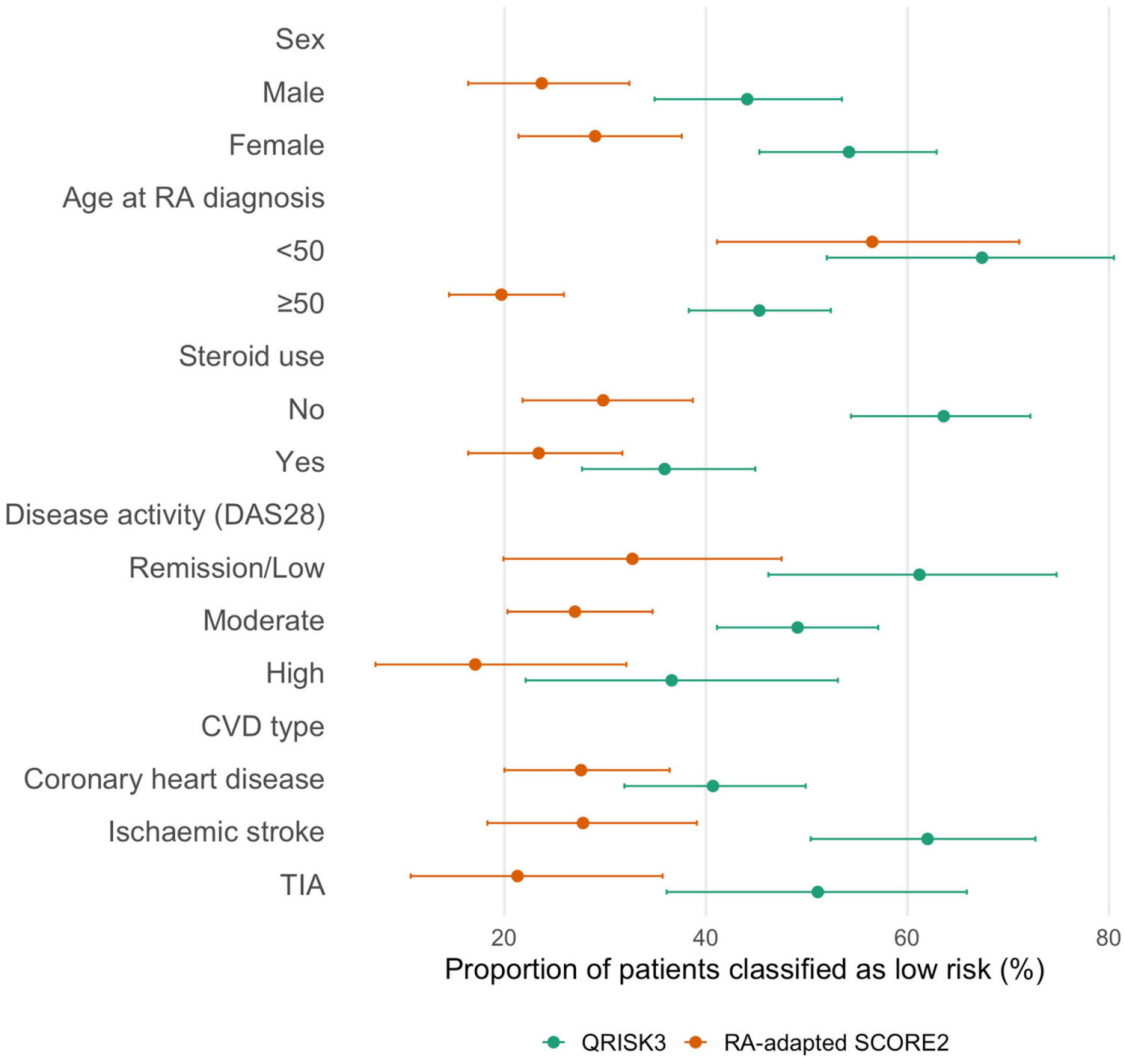
Risk underestimation by QRISK3 and RA-adapted SCORE2 across subgroups. Points represent proportions and horizontal bars indicate 95% confidence intervals. Subgroup sample sizes were: sex (female *n* = 131, male *n* = 118); age at RA diagnosis (<50 years *n* = 46, ≥50 years *n* = 203); steroid use (no *n* = 121, yes *n* = 128); disease activity by DAS28 (remission/low *n* = 49, moderate *n* = 159, high *n* = 41); and cardiovascular event type (coronary heart disease *n* = 123, ischaemic stroke *n* = 79, transient ischaemic attack *n* = 47).

Among patients diagnosed with RA before the age of 50 years, QRISK3 classified only 11 (23.9%) as high risk, with 22 (47.8%) misclassified as low risk by both tools. In contrast, patients diagnosed at age ≥50 were more frequently identified as high risk by both algorithms. Steroid use also influenced classification: 61% of glucocorticoid users were identified as high risk by both tools, compared with only 35% among non-users. By disease activity, patients with high DAS28 scores were most frequently classified as high risk (63.4% by both tools), while discordance was greatest in the moderate disease group. Differences were also observed by CVD subtype, with RA-adapted SCORE2 more frequently identifying patients with ischemic stroke as high risk compared with QRISK3. When stratified by time to cardiovascular event, QRISK3 underestimated risk in 43.6% (95% CI: 36.2–51.2) of patients who developed CVD within 10 years of RA diagnosis, increasing to 64.3% (95% CI: 51.9–75.4) among those with events occurring after 10 years; corresponding estimates for RA-adapted SCORE2 were 20.7% (95% CI: 15.0–27.3) and 41.4% (95% CI: 29.8–53.8), respectively. All subgroup results are reported in Figure 2 and Table 2.

**Table 2:** QRISK3 and RA-adapted SCORE2 classification within subgroups.

Subgroup	Category	Both high *n* (%)	QRISK3 high only *n* (%)	RA-adapted SCORE2 high only *n* (%)	Both low *n* (%)
Sex	Female	58 (44.3)	2 (1.5)	35 (26.7)	36 (27.5)
	Male	62 (52.5)	4 (3.4)	28 (23.7)	24 (20.3)
Age at RA diagnosis	<50 years	11 (23.9)	4 (8.7)	9 (19.6)	22 (47.8)
	≥50 years	109 (53.7)	2 (1.0)	54 (26.6)	38 (18.7)
Steroid use	No	42 (34.7)	2 (1.7)	43 (35.5)	34 (28.1)
		Yes	78 (60.9)	4 (3.1)	20 (15.6)
DAS28 category	Remission/Low	19 (38.8)	0	14 (28.6)	16 (32.7)
	Moderate	75 (47.2)	6 (3.8)	41 (25.8)	37 (23.3)
	High	26 (63.4)	0	8 (19.5)	7 (17.1)
CVD type	Coronary heart disease	69 (56.1)	4 (3.3)	20 (16.3)	30 (24.4)
	Ischemic stroke	28 (35.4)	2 (2.5)	29 (36.7)	20 (25.3)
	TIA	23 (48.9)	0	14 (29.8)	10 (21.3)

In multivariable analyses adjusted for age and sex (Table 3), several predictors of risk underestimation were identified. For QRISK3, patients with traditional CVD risk factors, like diabetes (OR, 0.04; 95% CI, 0.02–0.10), hyperlipidaemia (OR, 0.33; 95% CI, 0.14–0.74), and chronic kidney disease (OR, 0.07; 95% CI, 0.02–0.22), were associated with a lower likelihood of underestimation. Similar patterns were observed for RA-adapted SCORE2, with high disease activity (OR, 0.27; 95% CI, 0.07–0.96) linked to underestimation, whereas diabetes (OR, 0.01; 95% CI, 0.00–0.04) was strongly protective. The crude regression results are reported in Supplementary Table S1.

**Table 3:** Adjusted regression of factors associated with risk underestimation.

Factor	Underestimation in QRISK3	Underestimation in RA-adapted SCORE2	Underestimation in both tools
DAS28 (Ref: Remission/Low)			
Moderate	0.65 (0.31–1.38), *p* = 0.268	1.02 (0.43–2.51), *p* = 0.969	0.74 (0.31–1.78), *p* = 0.490
High	0.26 (0.09–0.69), *p* = 0.008	0.27 (0.07–0.96), *p* = 0.053	0.28 (0.07–0.96), *p* = 0.051
Extra-articular disease	1.10 (0.52–2.32), *p* = 0.808	0.71 (0.23–1.97), *p* = 0.532	0.85 (0.28–2.34), *p* = 0.769
Erosive disease	0.79 (0.39–1.59), *p* = 0.515	1.08 (0.46–2.46), *p* = 0.850	1.04 (0.44–2.35), *p* = 0.926
RA duration (per year)	1.02 (0.94–1.11), *p* = 0.674	0.99 (0.89–1.10), *p* = 0.869	0.99 (0.89–1.09), *p* = 0.817
Hypertension	1.11 (0.60–2.09), *p* = 0.736	0.49 (0.20–1.10), *p* = 0.094	0.63 (0.27–1.40), *p* = 0.269
Diabetes	0.04 (0.02–0.10), *p* < 0.001	0.01 (0.00–0.04), *p* < 0.001	0.01 (0.00–0.07), *p* < 0.001
Obesity	2.14 (0.70–7.35), *p* = 0.199	0.46 (0.12–1.63), *p* = 0.250	0.64 (0.16–2.18), *p* = 0.491
Hyperlipidaemia	0.33 (0.14–0.74), *p* = 0.009	0.44 (0.15–1.19), *p* = 0.121	0.45 (0.14–1.22), *p* = 0.134
Family history of CHD	0.59 (0.29–1.18), *p* = 0.139	0.45 (0.17–1.09), *p* = 0.089	0.59 (0.23–1.42), *p* = 0.259
Chronic kidney disease	0.07 (0.02–0.22), *p* < 0.001	0.34 (0.08–1.14), *p* = 0.101	0.02 (0.00–0.18), *p* = 0.005
Chronic lung disease	0.68 (0.31–1.44), *p* = 0.315	0.53 (0.18–1.39), *p* = 0.211	0.42 (0.14–1.14), *p* = 0.104

## Discussion

This study provides one of the evaluations of QRISK3 and RA-adapted SCORE2 in a Chinese cohort of patients with rheumatoid arthritis and established cardiovascular disease. This threshold-based approach reflects real-world use of cardiovascular risk calculators, which guide preventive treatment decisions based on predefined cut-offs rather than continuous risk estimates. We found that both tools substantially underestimated cardiovascular risk, with underestimation more pronounced for QRISK3 than RA-adapted SCORE2. Agreement between the two algorithms was only moderate, and discordance was particularly evident across age groups, steroid use, and disease activity categories. These findings suggest that existing RA-adapted cardiovascular risk models, which were developed and validated in European primary care populations, may not adequately capture the risk profile of Asian patients with RA. By directly comparing two widely used algorithms against observed cardiovascular outcomes, our study highlights the need for better-calibrated risk prediction tools tailored to Asian populations.

Our findings are in keeping with earlier work showing that conventional risk factors do not fully explain the excess cardiovascular burden in rheumatoid arthritis. Even when smoking, hypertension, and diabetes are accounted for, patients with RA continue to experience excess events, pointing to the role of systemic inflammation, cumulative glucocorticoid exposure, and disease activity as additional drivers (14). Existing tools attempt to address this in different ways: QRISK3 incorporates RA as a binary variable, while RA-adapted SCORE2 applies a simple multiplier. Neither approach, however, reflects the heterogeneity of RA severity or treatment histories, which likely contributes to the underestimation we observed. Prior studies that attempted to integrate inflammatory markers such as CRP into general population algorithms reported little improvement in discrimination, underscoring the difficulty of operationalising RA-specific risk in prediction models (15, 16). Furthermore, active systemic inflammation accelerates atherosclerosis and contributes to excess cardiovascular morbidity in RA (17). Our analysis found that patients with high DAS28 scores were more likely to underestimate their cardiovascular risk. Although our analyses relied on baseline disease activity at the time of diagnosis, remission is the primary target of modern RA management, and many patients subsequently achieve lower disease activity with treatment. Nonetheless, our results highlight that disease activity at presentation remains an important signal of cardiovascular risk, and that patients with uncontrolled disease may require closer monitoring beyond what current calculators provide. Nevertheless, because disease activity was assessed using a single baseline DAS28 measurement, our analysis does not capture cumulative or time-varying inflammatory burden. As sustained inflammation over time is a key determinant of cardiovascular risk in rheumatoid arthritis, reliance on baseline DAS28 may underestimate the contribution of inflammation to cardiovascular risk misclassification.

Notably, several established cardiovascular risk factors, including diabetes, chronic kidney disease, and hyperlipidaemia, were associated with a lower likelihood of risk underestimation. This pattern reflects how these variables are handled within the QRISK3 and RA-adapted SCORE2 algorithms rather than any protective biological effect. Because these conditions are explicitly incorporated and heavily weighted, their presence increases the probability that patients exceed predefined risk thresholds. Consequently, individuals with prominent traditional risk factors are less likely to be classified as low risk, even within a cohort restricted to patients who subsequently developed cardiovascular disease. In contrast, RA-related contributors such as disease activity and inflammatory burden are incompletely represented, leaving patients without major conventional risk factors particularly susceptible to risk underestimation.

Several mechanisms may further explain the systematic underestimation observed in our study. First, both QRISK3 and RA-adapted SCORE2 were developed and validated using composite outcomes that included fatal cardiovascular events. In contrast, our study was restricted to non-fatal endpoints. This definitional mismatch is important. Fatal and non-fatal events differ in both temporal occurrence and risk factor associations, and exclusion of mortality may partly explain the gap between predicted and observed risks. We did not capture cardiovascular death events in our study. Second, neither model perfectly captures key RA-specific contributors to cardiovascular risk. Chronic systemic inflammation, cumulative glucocorticoid exposure, and longer disease duration have consistently been linked with excess CVD risk in RA (18, 19). However, these features are either absent from RA-adapted SCORE2 or reduced to a single binary term in QRISK3. Third, differences in background population risk profiles are relevant. For example, smoking prevalence is substantially higher among Chinese men than in European populations (20). But lipid levels tend to be lower in Chinese patients, with both LDL-C and triglycerides typically below those observed in the Western population (21–23). Because both QRISK3 and RA-adapted SCORE2 place considerable weight on cholesterol measures, lower lipid concentrations may deflate predicted risk estimates despite high cardiovascular event rates. This discrepancy highlights how applying coefficients derived from Western cohorts may introduce systematic bias when used in Asian populations.

Future work should focus on recalibration of existing calculators using contemporary RA cohorts, ideally incorporating biomarkers of inflammation and longitudinal treatment data. Given population differences in risk factor profiles and background event rates, external validation across diverse healthcare settings, including Asian populations, is essential before these tools can be confidently applied in routine care. Ultimately, the value of any prediction tool will depend not only on its statistical performance but also on its ability to guide timely and effective prevention in high-risk patients.

This study was conducted in a hospital-based rheumatology service, which inevitably shapes the case mix of patients observed. In routine clinical practice, patients followed in secondary or tertiary care tend to have more active disease, greater treatment complexity, and a higher burden of comorbidity than those whose RA is well controlled in the community. Many are referred because of persistent symptoms, treatment escalation, or complications, rather than stable disease alone. It is therefore likely that our cohort over-represents patients with more severe or difficult-to-control RA, in whom cardiovascular risk is already elevated. As a consequence, the degree of risk underestimation observed in this study may not directly reflect that seen in community-managed RA populations with milder disease. At the same time, this setting mirrors where cardiovascular risk assessment and preventive decisions are most often revisited in practice, suggesting that underestimation may be most clinically relevant in hospital-managed patients rather than in lower-risk community cohorts.

This study has several limitations. First, the relatively small sample size substantially limits statistical power, particularly for subgroup analyses. Second, the single-centre, hospital-based design restricts generalisability, as patients there often represent more severe or complex cases than those managed in community settings. Third, as all participants had experienced a cardiovascular event, we were unable to perform standard calibration analyses or evaluate discrimination in the general RA population; instead, we were limited to assessing relative underestimation and agreement between tools. Although this case-only design does not permit assessment of discrimination or calibration, it allows evaluation of potential underestimation at clinically relevant decision thresholds. Conceptually, if a substantial proportion of patients who subsequently experienced a cardiovascular event are classified below recommended high-risk thresholds, this suggests that the risk tool may underestimate risk in this population when applied in routine clinical practice. Fourth, we relied on cross-sectional baseline data without repeated measures of disease activity, inflammatory markers, or treatment exposure, which may underestimate the contribution of dynamic RA-related factors. Fifth, important unmeasured confounders, such as diet, socioeconomic status, physical activity, and cumulative glucocorticoid exposure, were not available and may have influenced risk classification. Another limitation is the lack of detailed longitudinal data on the duration, severity, and cumulative burden of both rheumatoid arthritis and major cardiovascular risk factors, including diabetes, hypertension, dyslipidaemia, chronic kidney disease, and smoking. Future studies with prospective follow-up and time-updated exposures are needed to better capture cumulative risk.

## Conclusion

In summary, we found that QRISK3 and RA-adapted SCORE2 systematically underestimated cardiovascular risk in Chinese patients with RA and established CVD, reflecting both limitations in model design and differences in population risk profiles. These findings caution against uncritical application of Western-developed calculators in Asian settings and emphasise the importance of integrating RA-specific disease features into future prediction models. For clinicians, risk calculators should be viewed as supportive tools rather than definitive guides, with preventive strategies informed by broader clinical judgment that considers inflammation, treatment exposure, and comorbidities. For researchers, the priority is to recalibrate or redesign models using contemporary RA cohorts that reflect local practice and patient characteristics. Only through this iterative process can prediction tools move beyond statistical metrics to meaningfully guide prevention in high-risk RA populations.

## Data Availability

The original contributions presented in the study are included in the article/Supplementary Material, further inquiries can be directed to the corresponding author.
