# Supplementary material for: Underestimation of cardiovascular risk by QRISK3 and RA-adapted SCORE2 in a Chinese rheumatoid arthritis population
Source: Front Cardiovasc Med. 2026 Feb 12;13:1712088. doi: 10.3389/fcvm.2026.1712088 (PMC12935984; doi:10.3389/fcvm.2026.1712088)
Supplement: Supplementary file 1 [file Table1.docx]

Table S1: Crude regression of factors associated with risk underestimation

| **Factor** | **Underestimation in QRISK3** | **Underestimation in mSCORE** | **Underestimation in both tools** |
| --- | --- | --- | --- |
| **DAS28 (Ref: Remission/Low)** |  |  |  |
| Moderate | 0.61 (0.31–1.16) | 0.76 (0.39–1.55) | 0.63 (0.31–1.28) |
| High | 0.37 (0.15–0.85)* | 0.42 (0.15–1.13) | 0.42 (0.15–1.13) |
| **Extra-articular disease** | 0.83 (0.43–1.58) | 0.56 (0.23–1.23) | 0.65 (0.27–1.43) |
| **Erosive disease** | 1.08 (0.60–1.96) | 1.72 (0.90–3.23) | 1.65 (0.84–3.15) |
| **RA duration (per year)** | 1.16 (1.09–1.25)* | 1.19 (1.11–1.29)* | 1.18 (1.10–1.28)* |
| **RA diagnosis age (per year)** | 0.87 (0.83–0.91)* | 0.80 (0.74–0.85)* | 0.82 (0.76–0.87)* |
| **Hypertension** | 1.03 (0.59–1.80) | 0.54 (0.27–1.05) | 0.64 (0.31–1.25) |
| **Diabetes** | 0.10 (0.05–0.19)* | 0.02 (0.00–0.10)* | 0.02 (0.00–0.12)* |
| **Obesity** | 2.36 (0.90–6.93) | 0.99 (0.31–2.71) | 1.14 (0.35–3.12) |
| **Hyperlipidaemia** | 0.43 (0.21–0.87)* | 0.65 (0.27–1.43) | 0.62 (0.24–1.42) |
| **Family history of CHD** | 0.62 (0.34–1.13) | 0.60 (0.28–1.20) | 0.70 (0.32–1.41) |
| **Chronic kidney disease** | 0.13 (0.04–0.35)* | 0.52 (0.17–1.31) | 0.09 (0.01–0.45)* |
| **Chronic lung disease** | 0.70 (0.36–1.34) | 0.76 (0.34–1.58) | 0.63 (0.26–1.38) |
| **Regular steroid use** | 0.32 (0.19–0.53)* | 0.72 (0.41–1.27) | 0.65 (0.36–1.17) |
| **Sex (Ref: Female)** | 0.67 (0.40–1.10) | 0.76 (0.43–1.34) | 0.67 (0.37–1.21) |

*P<0.05
